# Assessment of trace elements pollution in the sea ports of New South Wales (NSW), Australia using oysters as bioindicators

**DOI:** 10.1038/s41598-018-38196-w

**Published:** 2019-02-05

**Authors:** Sayka Jahan, Vladimir Strezov

**Affiliations:** Department of Environmental Sciences, Faculty of Science and Engineering, Macquarie University NSW, 2109 Sydney, Australia

## Abstract

In this study Sydney rock oysters (*S*. *glomerata*) from six major sea ports of NSW, Australia were used as bioindicators to assess the distribution and levels of trace element accumulation in the ports. Substantial enrichment of Cu, Pb and Zn in the oysters of the sea ports were detected when compared to their background samples and the US Environmental Protection Agency (USEPA) provisional tolerable intake standard. Enrichment of As, Al, Fe, Mn, Br, Sr were also found in the oysters at the port areas. The bioconcentration ratios of the trace elements illustrated significant Fe, Cu, Zn, As, Mn, Al, Pb and Cr accumulation in *S*. *glomerate*. The biota sediment accumulation factor suggested Cu, Mn and Zn accumulation at two of the ports (Port Yamba and Botany), indicating availability of these metals in the oysters as strong metal accumulators. In addition, integrated metal contamination illustrated notable Fe, Zn, Cu and Al contamination at port environment, whereas cluster analysis portrayed interconnection between the contaminants and the study sites.

## Introduction

Trace element contamination is considered as one of the major issues in marine and estuarine environment due to their diverse sources, persistence, bioaccumulation, non-degradability and harmful effects on biota^1–7^. The ecological status of the aquatic environment can be evaluated by analyzing the distribution of trace elements in water, sediments and marine organisms^8^. In most cases, contaminated site assessment typically demands analysis of water and sediments to measure total trace elements concentrations, but often this is not a sufficient predictor of trace element toxicity to biota^9,10^. To overcome the problem, biomonitoring offers advantage as marine organisms’ (oysters, mussels, and clams) manifest greater spatial tolerant to elemental toxicity compared to water and sediments and therefore gained universal acceptance as the most reliable medium to ascertain sources of biologically available trace element contamination^6,8,11^. However, bivalve mollusks are considered to be one of the best bioindicators for coastal pollution studies due to their specific life traits, such as a sessile and filter-feeding behavior, a wide geographical distribution, abundance, sedentary and a relative resilience to pollutants^6,12^. Moreover, mollusc bivalves have the potential to accumulate chemical compounds at levels of 10^3^ at 10^5^ times more than other species^13^. Hence, since 1970’s a Worldwide scheme for monitoring ocean health by using mussels and oysters has been introduced^14–17^.

The specific ability of oysters to accumulate pollutants makes them candidate species for biomonitoring contaminant exposure to their potential biological effects^6,12,18,19^. Furthermore, oysters are often used as sentinel organisms due to their rapid adaptive capacity to the new environment. In Australia an integrated approach, including analysis of oysters as bioindicator and for quantification of elements in biota, was analyzed to monitor the impact of trace elements on port ecosystems^20,21^. As a common food source, it also urges investigation of the impact of marine activities on trace elements pollution in oysters.

Australian sea ports, which accommodate industry, commerce, tourism and recreation, often exacerbate trace elements contamination from different port related activities (transport and storage of hazardous materials, industrial installation, recreational shipping etc)^22–28^. This influences the growth rate and fecundity of marine biota and ultimately reduces the population diversity^26–31^ and reduces their suitability as a food source for humans^32^.

Among Pacific oysters, *S*. *glomerata* has been evidenced to be one of the most suitable organisms for biomonitoring chemical contamination in coasts and estuaries. This has preferentially been selected as a sentinel organism for its capability to concentrate pollutants, its lethargy, its limited ability to metabolize accumulated contaminants, its abundance, persistence, and ease of collection, all of which make it a good stable assimilator of the environment^33^. In the present study *S*. *glomerata* was used as the bioindicator to investigate the levels of trace element contamination in port environment, also applied by Goldberg *et al*.^34^ and Thompson *et al*.^35^. Evidence also suggested that *S*. *glomerata* is widely distributed species along the coastal belts of NSW that also acts as a potential accumulator of trace elements^35,36^.

The objective of this study was to assess the trace elements concentrations in the oysters of NSW sea ports to determine the variations of trace element bioaccumulation in the oysters under different port activities and to explore the level of trace element pollution in oysters which ultimately gives a scenario of stress on port environments. Finally, the present study typifies a new perspective for biomonitoring and risk assessment of trace elements in aquatic ecosystems using principal component and hierarchical cluster analysis methods.

## Materials and Methods

### Study area

**Figure 1:**
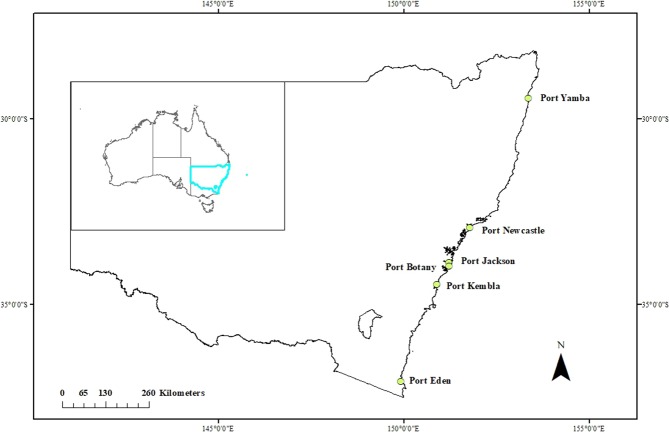
Map of the study area showing study ports of NSW.

The field study was conducted at the six major seaports in NSW, Australia, namely Port Jackson, Botany, Kembla, Newcastle, Yamba and Eden (Fig. 1). These ports are away from each other and are engaged with different shipping activities (23 km–1198 km). Port Jackson of Sydney harbour, which accommodates cruise shipping, pleasure boating and water sports, is a well-mixed estuary^37^. Port Botany is another important port of Sydney mainly engaged with shipping of containers, cruide oil, fossil fuel, chemicals and bio-fuels. Port Kembla is a prime export location for coal, grain terminal, bulk liquids, oil, fertiliser, pulp and steel products. The port is important for export and import of different mineral ores and petroleum products. Port Newcastle is the world largest port for coal export by tonnage that is also engaged with export and import of raw materials for steelworks, fertiliser and aluminium industries, grain, steel products, mineral sands and woodchips. Port Yamba is the eastern most sea port of New South Wales located at the mouth of the Clarence River. It is the second largest fishing port of New South Wales dealing with container liquid berth-livestock and explosive products. The Port of Eden is located in the South Coast region of New South Wales, Australia. The Port is the largest fishing port of New South Wales also engaged with export and import of woodchips, break bulk, machinery and equipment for the oil and gas industry^22,38^. The study locations are shown in Fig. 1.

### Sample collection and processing

Oyster samples known as Sydney rock oyster (*S*. *glomerata)* of different shell sizes (3 cm–7 cm) were collected from the six sea ports from April–June 2017. Three sampling points from each port were selected to collect samples among which one is background point selected from the same hydrogeological area but away from any influence of the port and other industrial activities. In this study, >40 indigenous oysters from each sampling point were collected by hand from dock columns and rocks in surface water (0–1 m). Immediately after collection, the oysters were stored in bags in a cooler box with ice and transported to the laboratory. About 20 oyster samples were selected from each sampling point and were weighed, and their tissues and shells were separateted. The tissues were then dried in an oven at 105 ± 5 °C for 8 hours to a constant weight^39^. The soft tissue, after the removal of the liquid, was then weighted. Prior to analysis, the dried samples were ground and the powdered sample then used for analysis where each analysis was replicated twice.

### Analytical procedure

The oyster tissue samples (0.05 g) were digested in 1 mL concentrated HNO_3_ acid at 80 °C on the hot plate for 24 hours until the samples were completely digested. The sample solutions were then diluted three times with Milli-Q water. Metals and major element (tweenty three) concentrations^21^ in the samples were determined by inductively coupled plasma mass spectrometry (ICP-MS Agilent 7700X and Varian vista-pro ICP-AES) respectively, while mercury was determined by cold vapour atomic absorption spectroscopy (CV-AAS) to reach the PQLs (practical quantitation limits). Quality and accuracy of the experimental procedure and the equipment was ensured using replicate analyses, certified referennce material (CRM) (oyster tissue, SRM 1566b) and sample spikes. The recovery percentage of all trace metals in CRM were 90–110% and the analytical precision expressed as coefficients of variance was <10% for all the metals based on replicate analysis. The detection limit of the method (MDL) was estimated as the standard error of 10 blank replicates^40^. The recovery percentage and detection limits of all trace elements are presented in Table 1.

**Table 1:** Recovery (%) and practical quantification limit (mg/kg dry wt.) of analyzed trace elements.

Trace elements	Recovery (%)	Detection limit (mg/kg dry wt.)
Al	96.3	0.99
As	102	3.99
Cr	105	0.09
Cu	103	0.09
Fe	108	0.99
Mn	102	0.99
Pb	95.6	0.99
Zn	101	0.09
Hg	98.3	0.1
Cd	106	0.4
Br	104	3.99
Si	99.8	9.99
Sr	109	0.99
Ti	106	0.99

### Data Processing

#### Bioconcentration Ratio (BCR)

Bioconcentration is a process in which biological organisms absorb a chemical compound from their surrounding environment through different body parts^41^. It is a quantitative measure of the biota’s bioaccumulative capacity^42^. The measured bioconcentration ratios also form the base for assessing the risk of adverse effects of hazardous substances on specific biota^43^. The extent of bioconcentration is calculated by using the formula (1)^44^:

1 $$BCR=\frac{{C}_{Organism}}{{C}_{Water}}$$

where C_organism_ is the concentration (mg/kg) of an element in the oyster, which was measured in this study, while C_Water_ is the concentration (mg/l) of the same element in the water of the same study locations, which was derived from the mean values published by Jahan and Strezov^37^. When the BCR is >1, bioaccumulation is considered.

#### Biota sediment accummulation factor (BSAF)

Biota sediment accumulation factor (BSAF) is the ratio between the concentration of element in a biota to the concentration of same element in sediment^45^. The BSAF for each element in the sample is calculated with equation (2):

2 $$BSAF=\frac{{C}_{organism}}{{C}_{Sediment}}$$

where C_organism_ and C_sediment_ are the concentrations(mg/kg) of trace elements in the oyster and in sediment^46^. Typically, BSAF value >1 indicates bioaccumulation of trace element. In this study, the sedimentary trace element concentrations for the same study locations were derived from the mean values published by Jahan and Strezov^22^.

#### Integrated metal contamination (IMC)

The severity of metal pollution can be determined using the integrated metal contamination equation (3) given by Liu and Wang^47^.

3 $$IMC=\sum _{i=0}^{m}{C}_{Contaminated}^{i}-{C}_{Clean}^{i}$$

where C^i^_Contaminated_ is the concentration(mg/kg) of i metal in a contaminated oyster obtained from the port area, C^i^_clean_ is a reference value (mg/kg) for the i metal in oyster obtained from the background site of each port, while m is the number of metals investigated, which is m = 13 for this calculation.

### Statistical analysis

Statistical analysis was performed by using Microsoft excel and SPSS version 24. Analyzed metal concentrations were presented as normalized concentration for standardized weight and length. The normality distribution of data were tested by Kolmogorov-Smirnov test and then normalized and analyzed using the multivariate statistical tools principal component analysis (PCA) and hierarchical cluster analysis (HCA) using both cases and variables to develop groups and identify links between elements and sampling sites by dendrogram as described by Jahan and Strezov^22^. The PCA and HCA were used to indentify the possible sources of trace elements in the sediments and group them based on their similarities. For PCA, only PCs with eigenvalue >1 were retained and variables were centered as mean^48^. Varimax rotation was applied to component loadings greater than 0.5 to facilitate the interpretation of the outcomes^49^.

## Results and Discussion

Bioaccumulation pattern and normalized concentrations of trace elements (whose concentrations are significantly high) in the soft tissue of the oysters (30–40 g and 5–7 cm) (*Saccostrea glomerata*) are shown in Table 2. The concentrations of As in port Jackson, Botany, Kembla and Eden range from 5 to 9 mg/kg which are significantly higher than their background concentrations (1.88 mg/kg) in oysters of the NSW coast given by Scanes and Roach^50^, and are also higher than the standard quality guidelines for bivalve mollusks (4 mg/kg) given by FAO^51^. However, in Australia and New Zealand, the regulation appllied to seafood is related to inorganic arsenic. This is because marine organisms and plants, such as shellfish, molluscs and seaweed, can contain high levels of arsenic, but mostly in organic arsenosugar forms^52^.

**Table 2:** Comparison of the studied trace elements concentrations (normalized concentration mean ± SD. for 30–40 g standardized weight and 5–7 cm length) in S. *glomerata* with that of the maximum permissible limits set forth by various organizations.

Parameters	mg/kg dry wt.	Al	As	Cr	Cu	Fe	Mn	Pb	Zn	Bo	Si	Br	Sr	Ti
Port Jackson	Bg	14	7	Bd	16	240	3	2	43	5	40	53	14	Bd
	Study point	21 ± 7.2	8.5 ± 1	Bd	6 ± 6.5	560 ± 440	5.5 ± 3.8	2 ± 0	45 ± 12	4 ± 0	35 ± 7	53 ± 1.4	155 ± 162	Bd
Port Botany	Bg	17	5	Bd	1	510	Bd	Bd	26	4	40	87	14	Bd
	Study point	12 ± 4.9	5 ± 1	Bd	14 ± 14	160 ± 250	8	Bd	17 ± 6	5 ± 0	25 ± 7	60 ± 13.4	10 ± 2	Bd
Port Kembla	Bg	32	4	Bd	13	140	3	Bd	23	4	30	68	180	Bd
	Study point	68.5 ± 26.8	7.5 ± 2.5	2 ± 0	18 ± 5	790 ± 0	10 ± 4	7 ± 5	51 ± 2.8	4 ± 1.2	55 ± 7	64 ± 12.7	51 ± 49.5	13 ± 15.6
Port Newcastle	Bg	19	Bd	Bd	1	21	7	Bd	16	7	60	56	33	5
	Study point	15 ± 2.1	Bd	Bd	10.5 ± 5	70 ± 38	4.5 ± 1	Bd	35 ± 5	3 ± 0	25 ± 4.2	56 ± 2.8	51 ± 58	Bd
Port Yamba	Bg	70	Bd	Bd	Bd	45	10	Bd	11	Bd	50	64	16	Bd
	Study point	99 ± 82.7	Bd	Bd	40 ± 29.7	220	23.5 ± 5	Bd	13 ± 3.3	5 ± 1.4	65 ± 49.5	55 ± 2	7 ± 1.4	2 ± 1.7
Port Eden	Bg	65	10	Bd	13	250	2	Bd	12	5	50	Bd	140	2
	Study point	41 ± 15	8 ± 2	Bd	3 ± 1	260 ± 125	2.5 ± 1	Bd	45.5 ± 10.6	6 ± 2.8	40 ± 0	Bd	32 ± 21.9	1 ± 0
Background concentration^**a**^	NSW		1.9		21.6		2.5	0.09	277					
FAO 1989^b^			4		20			0.2						
US EPA 2013^c^	PTI				0.05			0.03	0.3–1					
US FDA 1993^d^				13				1.7						

Bg = background, Bd = below detection, dry wt. = dry weight, PTI = Provisional tolerable intake.

^a^Background concentration by Scanes & Roach^50^.

^b^Standard quality guidelines for bivalve mollusks (FAO, 1989).

^c^USEPA (2013).

^d^U.S Food and Drug Administration, 1993.

The Cu concentrations in the oyster of all ports were found to be higher than the USEPA (provisional tolerable intake)^53^ standard (0.05 mg/kg) and FAO standard quality guidelines for bivalve mollusks (20 mg/kg)^51^. The highest concentration of Cu was detected at port Yamba (61 mg/kg), which is significantly above the standards and is identified as unsafe food^54^. Cu enrichment in the oysters at port Botany, Kembla, Newcastle and Yamba comparing to their background sites advocates the impacts of port activities which are associated with trade of coal, steel products, crude oil, fossil fuels, chemicals, fertilizers, mineral sands, preservative chemicals from wood chips and storage of hazardous products in the port vicinity^37^. However, Cu concentrations found in oysters are also associated with higher assimilation efficiencies and bioavailability in the port environment. The normalized Pb concentrations in the studied *S*. *glomerata* species at port Kembla exhibited six-fold increase when compared to the maximum permissible limits recommended by Food and Drug Administration^55^ and recommended as unsafe for human consumption. The Pb concentration (2–7 mg/kg) in this port is also higher than its background site and all other standards (0.2 0.025 and 1.7 mg/kg by FAO, USEPA and USFDA respectively) shown in Table 2. The industrial complexes, including the major metal smelting operations adjoining this port are likely the major sources of Pb^56^. However, Zn concentrations (13–51 mg/kg) in the oyster at all ports also demonstrate the oysters are unsafe as food as their concentrations are considerably higher than their corresponding USEPA (provisional tolerable intake) standard (0.3–1 mg/kg). It is known that mollusks possess high affinity for accumulation of Zn^57^ and it is generally agreed that the highest concentrations of zinc in marine biota are found in the tissues of filter feeding mollusks, especially oysters^58^. Unlike port Eden, the oysters in all other ports contained higher concentrations of Mn than the background values given by Scanes and Roach^49^. The results also portray remarkable Mn bioaccumulation inside port Jackson, Botany, Kembla and Yamba comparing to their background sites. Notable amounts of Al, Fe, Br, Si and Sr were detected in almost all ports although they do not have any standard values to compare. Significant amounts of Ti is also found in the oysters at port Kembla. In addition, concentrations of some elements (Hg, Cd, Ag, Ni, Co, Ba, Sn) were measured but found bellow detection limit in all study points, therefore they were not reported further.

### Bioconcentration ratio (BCR)

**Figure 2:**
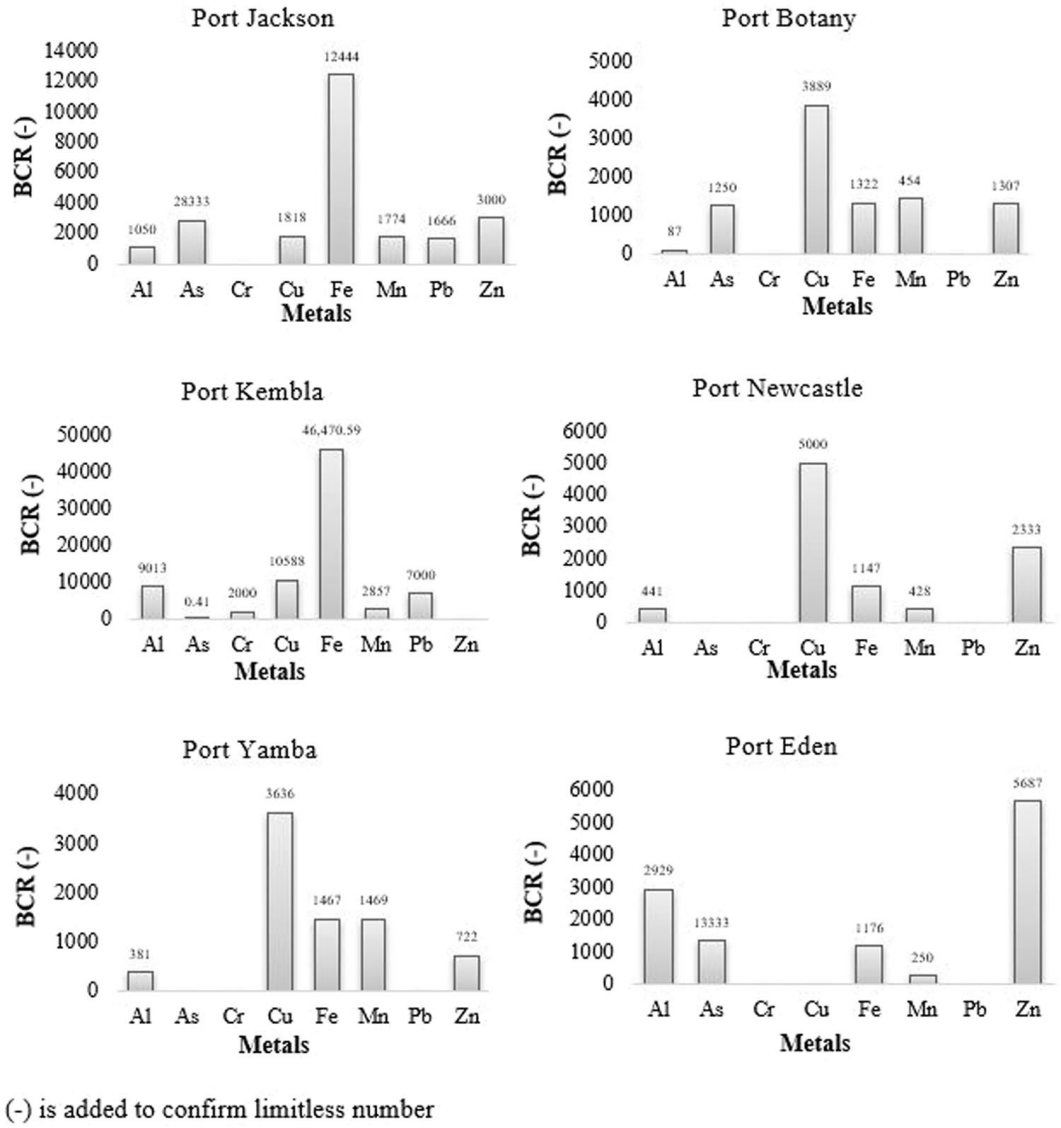
Bioconcentration Ratio (BCR) in oysters (S. *glomerata*) from the seaports of NSW, Australia.

BCR values in S. *glomerata* shown in Fig. 2 present an order of Fe > Cu > Zn > As > Mn > Al > Pb > Cr although they show variations among ports. BCR values of As, Cr and Pb in some of the ports are less than 1 which demonstrate almost similar concentrations of those elements in oyster and water. However, only eight metals are calculated because of others are below detection limit in water. BCR values greater than 1000 indicate significant and slow accumulation^59^. High BCR values also demonstrate the uptake of free metal ions from solution more effectively via dermal organs^60^. BCR values of Fe and Cu in almost all ports are >1000 with the highest values (Fe = 46,470, Cu = 10,588) at Port Kembla which demonstrate considerable Fe and Cu concentrations in the port environment. Except Port Kembla and Yamba, significant Zn concentrations (as BCR > 1000) were found in all other ports.

### Biota sediment accumulation factor (BASF)

**Figure 3:**
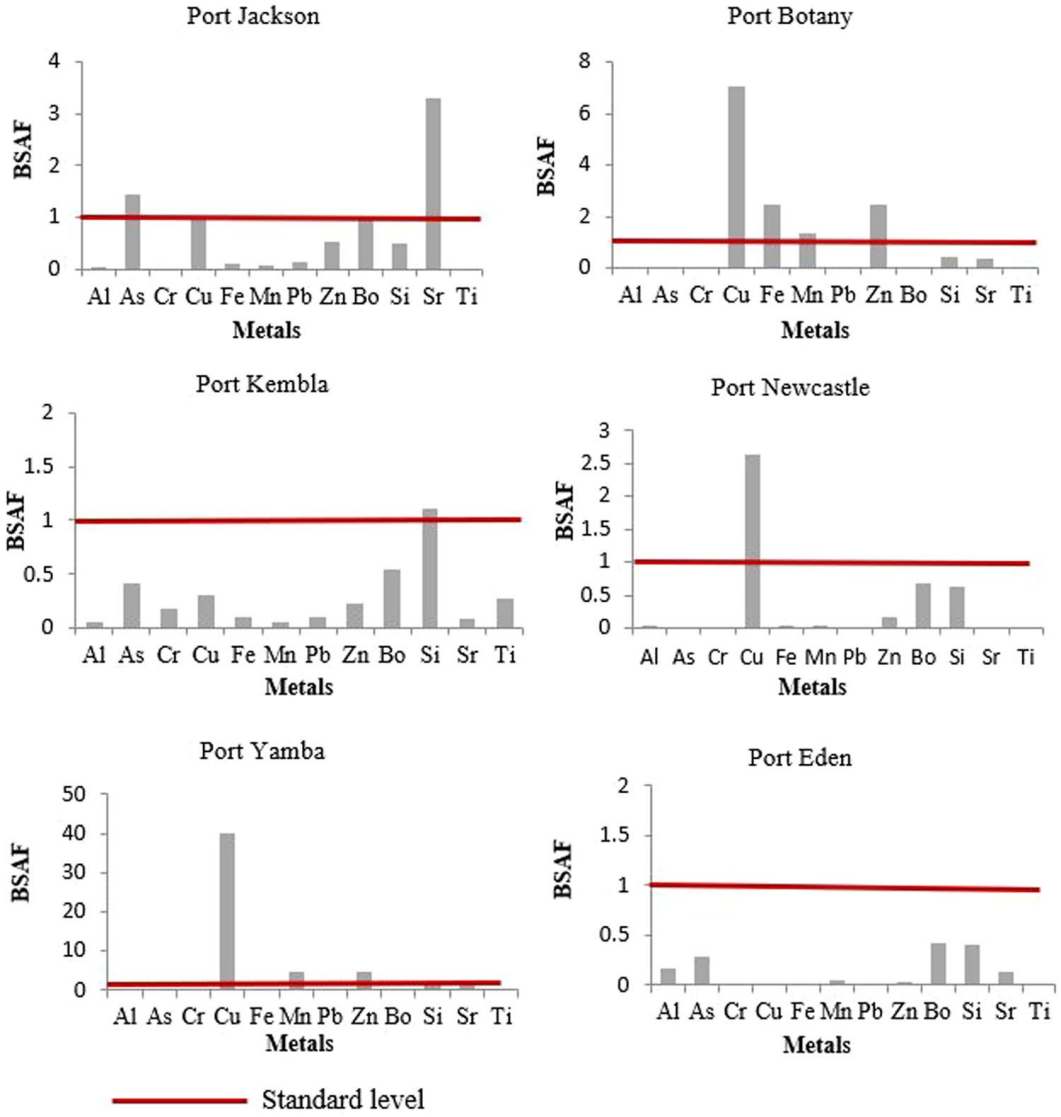
Biota sediment accumulation factors (BSAFs) for oysters in the study ports of NSW, Australia.

The average concentration of the trace element was then applied to determine the biota sediment accumulation factor, as presented in Fig. 3. Significant bioaccumulation of Cu at port Botany (7), Newcastle (2.63) and Yamba (40) and bioaccumulation of Zn (2.43 and 4.33) and Mn (4.70 and 1.33) at port Botany and Yamba indicates availability of these metals in the port environment as well as high-level absorbing capacity in the soft tissues of the oysters. Bioaccumulation of As and Sr were observed at port Jackson whereas Si bioaccumulation was also found in the oysters at port Kembla. Based on the results, S. *glomerata* is considered to be strong accumulators for Cu and moderate accumulators for Zn and Mn.

### Integrated metal concentration

The severity of metal pollution by integrated metal concentration (IMC) is presented in Table 3. The results suggest that the oysters at port Eden and Botany are comparatively less contaminated than the oyster samples from the other port sites. The results also imply that the oysters at port Kembla are severely contaminated followed by port Jackson and port Yamba with notable enrichment of Fe, Zn, Cu and Al. For calculation of IMC reference site values are required. If the reference values are affected by non-point pollution sources the IMC values may be affected because of the undue influence of one of the measurements used in the final composite values^61^. Therefore, no threshold for maximum pollution is given for this index.

**Table 3:** Integrated metal contamination (mg/kg) in the oysters of NSW seaports.

Port	Integrated metal concentration (mg/kg)								Total=
	Al	As	Cu	Fe	Mn	Zn	Sr	Ti	∑(C_contaminated_ − C_clean_)
Jackson	7	1.5	−10	320	2.5	2	141	0	458
Botany	−5	0	13	−305	8	−9	−4	0	−388
Kembla	36.5	3.5	5	650	7	28	−129	13	644
Newcastle	−4	0	9.5	49	−2.5	19	18	−5	45
Yamba	29	0	40	175	13.5	2	−9	2	264
Eden	−24	−2	−10	10	0.5	33.5	−108	−1	−110

The variations of trace elements concentrations in the oysters between the background and study port areas by means of ANOVA are shown in Table 4. The results revealed that the variations were insignificant (P > 0.05).

**Table 4:** Significance (variations were significant at P < 0.05) analysis of trace elements concentrations between the background and port oysters.

Study Area	Df	P-Value
Port Jackson	24	0.45
Port Botany	24	0.46
Port Kembla	24	0.42
Port Newcastle	24	0.7
Port Yamba	24	0.29
Port Eden	24	0.76

Correlation analysis was also performed on the normalized data set to test the relationship between the environmental parameters and significant correlations among metals are presented in Table 5. According to the Pearson statistical analysis (significant at P < 0.05) strong positive relationship exists between weight and length of oyster (r^2^ = 0.98). Al shows strong positive correlation with Cu, Mn and Si whereas Cr shows strong positive relation with Pb and Ti. However, Cu shows strong positive correlation with Mn and Si, while Mn is strongly correlated with Si and I. Analysis results also reveal that a strong positive correlation exists between Pb and Sr.

**Table 5:** Correlation analysis of trace elements in the oyster of the seaports of NSW, Australia.

	Length	Weight	Al	As	Cd	Cr	Cu	Fe	Mn	Ni	Pb	Zn	B	Si	Br	Sr	Ti	I
Length	1																	
Weight	**0**.987*	1.000																
Al	0.731	0.791	1.000															
As	−0.601	−0.666	−0.339	1.000														
Cd	−0.315	−0.277	0.069	0.610	1.000													
Cr	−0.125	−0.106	0.181	0.441	0.731	1.000												
Cu	0.605	0.681	**0**.863*	−0.557	−0.302	−0.004	1.000											
Fe	0.044	−0.069	0.060	0.739	0.359	0.477	−0.224	1.000										
Mn	0.726	0.765	**0**.931*	−0.401	−0.202	0.123	**0**.947*	0.073	1.000									
Ni	0.021	0.119	0.554	−0.129	0.078	0.447	0.748	−0.121	0.655	1.000								
Pb	−0.117	−0.130	0.131	0.540	0.682	**0**.980*	−0.068	0.632	0.104	0.365	1.000							
Zn	−0.241	−0.342	−0.398	0.749	0.578	0.369	−0.772	0.722	−0.538	−0.604	0.474	1.000						
B	−0.139	−0.063	0.355	0.268	0.520	−0.047	0.111	−0.047	0.083	0.106	−0.116	0.017	1.000					
Si	0.752	0.799	**0**.996*	−0.315	0.032	0.183	**0**.862*	0.125	**0**.949*	0.544	0.149	−0.374	0.303	1.000				
Br	0.104	0.097	0.079	−0.211	−0.327	0.392	0.385	0.059	0.372	0.586	0.408	−0.349	−0.736	0.121	1.000			
Sr	0.032	−0.129	−0.384	0.475	−0.132	0.001	−0.509	0.755	−0.268	−0.574	0.191	0.690	−0.464	−0.306	0.062	1.000		
Ti	−0.138	−0.118	0.177	0.458	0.759	**0**.999*	−0.022	0.478	0.105	0.432	**0**.977*	0.387	−0.014	0.176	0.354	−0.007	1.000	
I	**0**.900*	**0**.853*	0.712	−0.368	−0.401	−0.070	0.650	0.314	**0**.816*	0.156	0.000	−0.211	−0.215	0.765	0.292	0.249	−0.091	1.000

“Bold*” mark denotes strong correlation.

Principal component analysis (PCA) of the oyster data summarizes four groups of pollutants and the contamination levels of each group of pollutants in the oysters of the studied ports. Four significant principal component groups were determined by deriving the eigenvalues and eigenvectors from the correlation matrix. The percentage of the total variance of each principal component (PC) group is shown in Table 6. Four component groups generating about 95.8% of the total variance were obtained. The first component group consists of 37.75% of the variation with the greatest weights (>0.70) for Cu, Mn, U, Cd and Br, and moderate weights for I and Si. PC_2_ accounted for 27.64% of the variation with the important components comprising of Fe, Pb, Cr and Al. PC_3_ and PC_4_ exhibited 21.03% and 9.37% of the variation respectively and had moderate weights for U, Br and I.

**Table 6:** Component matrix of the oysters of NSW seaports.

	PC_1_	PC_2_	PC_3_	PC_4_
Eigenvalues % of variance Cumulative %	5.66	4.15	3.16	1.40
	37.75	27.64	21.03	9.37
	37.75	65.39	86.42	95.80
**Eigenvectors**				
Cu	0.94	0.136	−0.127	−0.282
Mn	0.887	0.439		
U	0.783		0.602	−0.133
Br	0.769		0.603	−0.199
Zn	−0.754	0.385	0.462	0.232
Cd	0.707	−0.536	0.333	0.224
I	0.688	0.330	0.114	0.636
As	−0.641	0.457	0.174	−0.211
Fe	−0.184	0.866	0.145	0.36
Pb		0.819	0.464	−0.328
Cr		0.77	0.301	−0.509
Al	0.392	0.735	−0.509	0.147
Si	0.562	0.667	−0.431	0.203
Bo	−0.27	0.362	−0.858	
Sr	−0.507	0.23	0.732	0.384

Extraction Method: Principal Component Analysis.

^a^4 components extracted.

**Figure 4:**
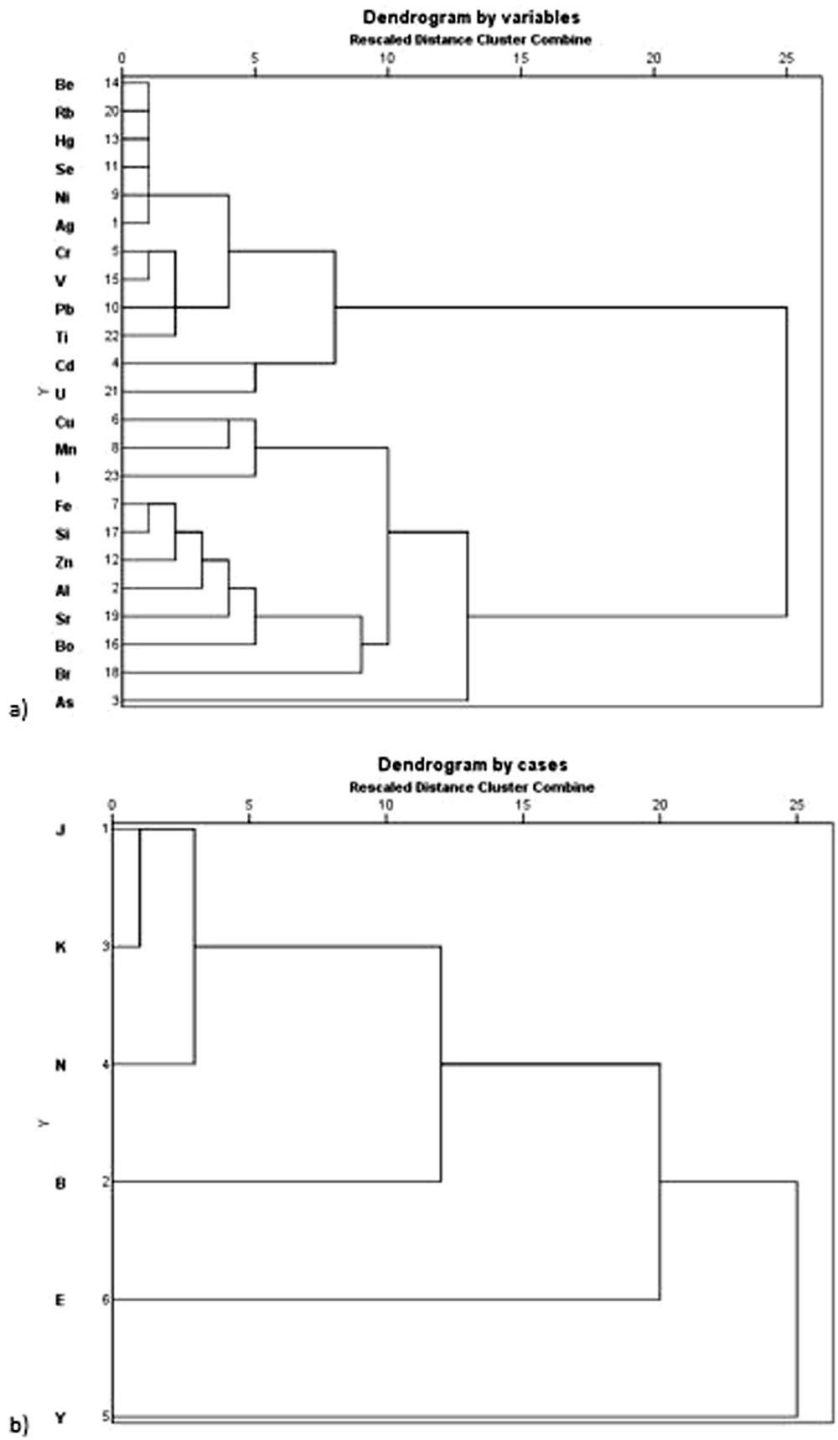
Hierarchical dendrogram showing the clustering of (**a**) trace elements and (**b**) study sites of the sea ports of NSW, Australia.

The cluster analysis (HCA) results for the sampling sites based on the trace element concentrations were presented as a dendrogram shown in Fig. 4. Two main different clusters were identified from the trace element enrichment dendrogram (Fig. 4a). The first cluster group comprising Se, Cd, U, Cr, V, Pb and Ti with two sub-groups of Se, Cd, U as one sub-group and Cr, V, Pb and Ti as the second sub-group. The second HCA cluster group also consists of two sub-cluster groups, one of which comprises of Cu, Mn and I and the other group includes Fe, Si, Zn, Al, B, Sr, Br and As.

The dendrogram can also help to explain and group the impact of port activities on trace element enrichments, as presented in Fig. 4b. The analysis results demonstrated that the fishing fleet activities and trade of woodchip, break and bulk machinery for the oil and gas industry at port Eden are significantly responsible for the trace element contamination in oyster followed by the container, cruide oil and bulk liquid operations (fossil fuel, chemical and bio-fuel) at port Botany and bulk liquids, oil, fertiliser, pulp, steel products and various ores related activities at port Kembla.

## Conclusion

The present study showed the pattern distribution of trace elements in the sea port environments using oyster (*S*. *glomerata*) as a bioindicator. *S*. *glomerata* has been known as an effective ecological tool to trace the heavy metals or toxic elements (for example, Cu, Zn, As, Pb, Fe, Mn and Sr) as it is widely grown in the Pacific coastal areas. The results illustrate that the varying levels of trace elements in the oyster and their concentrations were highly dependent on the nature of the ports and human activities in the vicinity of the port areas. The BCR and BSAF analyses demonstrate significant accumulation of Fe, Cu, Mn, Zn, As and Sr, which reflect their availability in seawater and sediments. Likewise, the integrated metal contamination analysis determined severe contamination of Fe, Zn, Cu and Al contamination in the oysters at all port areas. Overall, *S*. *glomerata* is an important bioindicator to detect the distribution of contaminants in the port environment. Further measures are still required for suitable and effective management of the toxic trace elements in the NSW ports to alleviate the anthropogenic impacts on the sea environment.

## Supplementary information

Supplementary dataset 1

## Data Availability

The datasets generated during and/or analyzed during the current study are available in supplementary dataset.
