# Supplementary material for: Assessment of trace elements pollution in the sea ports of New South Wales (NSW), Australia using oysters as bioindicators
Source: Sci Rep. 2019 Feb 5;9:1416. doi: 10.1038/s41598-018-38196-w (PMC6363737; doi:10.1038/s41598-018-38196-w)
Supplement: Supplementary file 1 — Supplementary dataset 1 [file 41598_2018_38196_MOESM1_ESM.pdf]

Assessment of trace elements pollution in the sea ports of New South Wales (NSW), Australia using oysters as bioindicators

Sayka Jahan* and Vladimir Strezov

BSAF

Parameters	mg/kg	Al Biota	Al Sedi	BSAF	As Bio	As Sedi	BSAF	Cr Bio	Cr Sedi	BSAF	Cu Bio	Cu Sedi	BSAF	Fe Bio	Fe Sedi	BSAF	Mn Bio	Mn Sedi	BSAF
**Port Jackson**	Study point	21.00	895.00	0.02	8.50	6.00	1.42	0.00	4.00	0.00	6.00	6.00	1.00	560.00	7300.00	0.08	5.50	110.00	0.05
**Port Botany**	Study point	12.00	930.00	0.01	5.00	0.00	0.00	0.00	2.00	0.00	14.00	2.00	7.00	160.00	765.00	0.21	8.00	6.00	1.33
**Port Kembla**	Study point	68.50	1250.00	0.05	7.50	18.50	0.41	2.00	11.00	0.18	18.00	59.00	0.31	790.00	8000.00	0.10	10.00	201.00	0.05
**Port Newcastle**	Study point	15.00	1550.00	0.01	0.00	4.00	0.00	0.00	4.00	0.00	10.50	4.00	2.63	70.00	5300.00	0.01	4.50	185.00	0.02
**Port Yamba**	Study point	99.00	383.00	0.26	0.00	0.00	0.00	0.00	1.00	0.00	40.00	1.00	40.00	220.00	760.00	0.29	23.50	5.00	4.70
**Port Eden**	Study point	41.00	2250.00	0.02	8.00	29.00	0.28	0.00	31.00	0.00	3.00	1195.00	0.00	260.00	46000.00	0.01	2.50	73.00	0.03

Parameters	mg/kg	Pb Bio	Pb Sedi	BSAF	Zn Bio	Zn Sedi	BSAF	B Bio	B Sedi	BSAF	Si Bio	Si Sedi	BSAF	Sr Bio	Sr Sedi	BSAF	Ti Bio	Ti Sedi	BSAF
**Port Jackson**	Study point	2.00	18.00	0.11	45.00	85.00	0.53	4.00	4.00	1.00	35.00	75.00	0.47	155.00	56.00	2.77	0.00	56.00	0.00
**Port Botany**	Study point	0.00	2.00	0.00	17.00	7.00	2.43	5.00	0.00	0.00	25.00	60.00	0.42	10.00	28.00	0.36	0.00	17.00	0.00
**Port Kembla**	Study point	7.00	74.00	0.09	51.00	235.00	0.22	4.00	7.50	0.53	55.00	50.00	1.10	51.00	510.00	0.10	13.00	47.00	0.28
**Port Newcastle**	Study point	0.00	24.00	0.00	35.00	78.00	0.45	3.00	4.50	0.67	25.00	240.00	0.10	51.00	80.00	0.64	0.00	37.00	0.00
**Port Yamba**	Study point	0.00	1.00	0.00	13.00	3.00	4.33	5.00	0.00	0.00	65.00	45.00	1.44	7.00	4.50	1.56	2.00	10.00	0.20
**Port Eden**	Study point	0.00	165.00	0.00	45.50	2345.00	0.02	6.00	14.50	0.41	40.00	100.00	0.40	32.00	245.00	0.13	1.00	73.00	0.01

$$BSAF = \frac{C_{Organism}}{C_{Sediment}}$$

BCF

Parameters	mg/kg	Al Biota	Al water	BCF	As Bio	As water	BCF	Cr Bio	Cr water	BCF	Cu Bio	Cu wate	BCF
**Port Jackson**	Study point	21.000	0.020	1050.00	8.500	0.003	2833.33	0.00	4.00	0.00	6.00	0.003	1818.18
**Port Botany**	Study point	12.000	0.137	87.59	5.000	0.004	1250.00	0.00	2.00	0.00	14.00	0.004	3888.89
**Port Kembla**	Study point	68.500	0.008	9013.16	7.500	18.500	0.41	2.00	0.00	2000.00	18.00	0.002	10588.24
**Port Newcastl**	Study point	15.000	0.034	441.18	0.000	4.000	0.00	0.00	4.00	0.00	10.50	0.002	5000.00
**Port Yamba**	Study point	99.000	0.260	380.77	0.000	0.000	0.00	0.00	1.00	0.00	40.00	0.011	3636.36
**Port Eden**	Study point	41.000	0.014	2928.57	8.000	0.006	1333.33	0.00	31.00	0.00	3.00	0.000	0.00

Parameters	mg/kg	Fe Bio	Fe water	BCF	Mn Bio	Mn wate	BCF	Pb Bio	Pb water	BCF	Zn Bio	Zn wate	BCF
**Port Jackson**	Study point	560.00	0.05	12444.44	5.50	0.003	1774.19	2.00	0.001	1666.67	45.00	0.015	3000.00
**Port Botany**	Study point	160.00	0.12	1322.31	8.00	0.006	1454.55	0.00	0.002	0.00	17.00	0.013	1307.69
**Port Kembla**	Study point	790.00	0.02	46470.59	10.00	0.004	2857.14	7.00	0.001	7000.00	51.00	0.000	0.00
**Port Newcastl**	Study point	70.00	0.06	1147.54	4.50	0.011	428.57	0.00	24.000	0.00	35.00	0.015	2333.33
**Port Yamba**	Study point	220.00	0.15	1466.67	23.50	0.016	1468.75	0.00	1.000	0.00	13.00	0.018	722.22
**Port Eden**	Study point	260.00	0.22	1176.47	2.50	0.010	250.00	0.00	165.000	0.00	45.50	0.008	5687.50

	Al	As	Cr	BCF<br>Cu	Fe	Mn	Pb	Zn
**Port Jackson**	1050.00	2833.33	0.00	1818.18	12444.44	1774.19	1666.67	3000.00
**Port Botany**	87.59	1250.00	0.00	3888.89	1322.31	1454.55	0.00	1307.69
**Port Kembla**	9013.16	0.41	2000.00	10588.24	46470.59	2857.14	7000.00	0.00
**Port Newcastl**	441.18	0.00	0.00	5000.00	1147.54	428.57	0.00	2333.33
**Port Yamba**	380.77	0.00	0.00	3636.36	1466.67	1468.75	0.00	722.22
**Port Eden**	2928.57	1333.33	0.00	0.00	1176.47	250.00	0.00	5687.50

IMC

$$\text{Integrated metal contamination} = \sum_{i=0}^{m} C^{i}_{Contaminated} - C^{i}_{Clean}$$

**Parameters**	**Port Jackson**		**IMC**	**Port Botany**		**IMC**	**Port Kembla**		**IMC**	**Port Newcastle**		**IMC**	**Port Yamba**		**IMC**	**Port Eden**		**IMC**
**mg/kg**	Bg	Study point		Bg	Study point		Bg	Study point		Bg	Study point		Bg	Study point		Bg	Study point	
**Al**	14	21	7	17	12	-5	32	68.5	36.5	19	15	-4	70	99	29	65	41	-24
**As**	7	8.5	1.5	5	5	0	4	7.5	3.5	0	0	0	0	0	0	10	8	-2
**Cr**	0	0	0	0	0	0	0	2	2	0	0	0	0	0	0	0	0	0
**Cu**	16	6	-10	1	14	13	13	18	5	1	10.5	9.5	0	40	40	13	3	-10
**Fe**	240	560	320	510	160	-350	140	790	650	21	70	49	45	220	175	250	260	10
**Mn**	3	5.5	2.5	0	8	8	3	10	7	7	4.5	-2.5	10	23.5	13.5	2	2.5	0.5
**Pb**	2	2	0	0	0	0	0	7	7	0	0	0	0	0	0	0	0	0
**Zn**	43	45	2	26	17	-9	23	51	28	16	35	19	11	13	2	12	45.5	33.5
**Bo**	5	4	-1	4	5	1	4	4	0	7	3	-4	0	5	5	5	6	1
**Si**	40	35	-5	40	25	-15	30	55	25	60	25	-35	50	65	15	50	40	-10
**Br**	53	53	0	87	60	-27	68	64	-4	56	56	0	64	55	-9	0	0	0
**Sr**	14	155	141	14	10	-4	180	51	-129	33	51	18	16	7	-9	140	32	-108
**Ti**	0	0	0	0	0	0	0	13	13	5	0	-5	0	2	2	2	1	-1
			458			-388			644			45			263.5			-110

	**Integrated metal concentration**								
Port	Al	As	Cu	Fe	Mn	Zn	Sr	Ti	$\sum(C_{contaminated} - C_{clean})$
Jackson	7	1.5	-10	320	2.5	2	141	0	458
Botany	-5	0	13	-305	8	-9	-4	0	-388
Kembla	36.5	3.5	5	650	7	28	-129	13	644
Newcastle	-4	0	9.5	49	-2.5	19	18	-5	45

Yamba	29	0	40	175	13.5	2	-9	2	263.5
Eden	-24	-2	-10	10	0.5	33.5	-108	-1	-110

Correlation

mg/kg	Length	Weight	Al	As	Cd	Cr	Cu	Fe	Mn	Ni	Pb	Zn	B	Si	Br	St	Ti	I
J	148	45	27	9	0	0	9	960	9	0	2	57	4	40	54	270	0	4
B	136	42	16	5	0	0	26	100	8	1	0	19	5	30	69	11	0	0
K	145	45	85	9	0.5	2	22	790	13	1	10	53	5	60	73	86	24	2
N	151	47	16	0	0	0	11	98	5	0	0	38	3	30	59	92	0	2
Y	161	51	180	0	0	0	61	310	27	1	0	16	6	100	56	8	0	6
E	142	44	45	8	0.4	0	4	380	3	0	0	53	8	40	0	48	1	0

	Length	*Weight*	*Al*	*As*	*Cd*	*Cr*	*Cu*	*Fe*	*Mn*	*Ni*	*Pb*	*Zn*	*B*	*Si*	*Br*	*St*	*Ti*	*I*
Length	1.00																	
Weight	0.99	1.00																
Al	0.73	0.79	1.00															
As	-0.60	-0.67	-0.34	1.00														
Cd	-0.32	-0.28	0.07	0.61	1.00													
Cr	-0.12	-0.11	0.18	0.44	0.73	1.00												
Cu	0.61	0.68	0.86	-0.56	-0.30	0.00	1.00											
Fe	0.04	-0.07	0.06	0.74	0.36	0.48	-0.22	1.00										
Mn	0.73	0.77	0.93	-0.40	-0.20	0.12	0.95	0.07	1.00									
Ni	0.02	0.12	0.55	-0.13	0.08	0.45	0.75	-0.12	0.66	1.00								
Pb	-0.12	-0.13	0.13	0.54	0.68	0.98	-0.07	0.63	0.10	0.37	1.00							
Zn	-0.24	-0.34	-0.40	0.75	0.58	0.37	-0.77	0.72	-0.54	-0.60	0.47	1.00						
B	-0.14	-0.06	0.36	0.27	0.52	-0.05	0.11	-0.05	0.08	0.11	-0.12	0.02	1.00					
Si	0.75	0.80	1.00	-0.31	0.03	0.18	0.86	0.13	0.95	0.54	0.15	-0.37	0.30	1.00				
Br	0.10	0.10	0.08	-0.21	-0.33	0.39	0.39	0.06	0.37	0.59	0.41	-0.35	-0.74	0.12	1.00			
St	0.03	-0.13	-0.38	0.48	-0.13	0.00	-0.51	0.75	-0.27	-0.57	0.19	0.69	-0.46	-0.31	0.06	1.00		
Ti	-0.14	-0.12	0.18	0.46	0.76	1.00	-0.02	0.48	0.11	0.43	0.98	0.39	-0.01	0.18	0.35	-0.01	1.00	
I	0.90	0.85	0.71	-0.37	-0.40	-0.07	0.65	0.31	0.82	0.16	0.00	-0.21	-0.22	0.77	0.29	0.25	-0.09	1.00
